# From Nutritional Profiles to Digestibility Insights: Exploring Palm Kernel Cake and Decanter Cake in Broiler Diets

**DOI:** 10.3390/ani15131966

**Published:** 2025-07-04

**Authors:** Mohammad Naeem Azizi, Teck Chwen Loh, Eric Lim Teik Chung, Muhamad Faris Ab Aziz, Hooi Ling Foo, Jiaxiang Liu, Zakaria Aiman Farzana, Letchumanan Samuel Raj

**Affiliations:** 1 Department of Animal Science, Faculty of Agriculture, Universiti Putra Malaysia (UPM), Serdang 43400, Selangor, Malaysia; naimazizi83@gmail.com (M.N.A.); ericlim@upm.edu.my (E.L.T.C.); mhd_faris@upm.edu.my (M.F.A.A.); liu253659786@163.com (J.L.); 210795@student.upm.edu.my (Z.A.F.);; 2 Department of Pre-Clinic, Faculty of Veterinary Science, Afghanistan National Agricultural Sciences and Technology University (ANASTU), Kandahar 3801, Afghanistan; 3 Institute of Tropical Agriculture and Food Security, Universiti Putra Malaysia (UPM), Serdang 43400, Selangor, Malaysia; 4 Department of Bioprocess Technology, Faculty of Biotechnology and Biomolecular Science, Universiti Putra Malaysia (UPM), Serdang 43400, Selangor, Malaysia; hlfoo@upm.edu.my; 5 Institute of Bioscience, Universiti Putra Malaysia (UPM), Serdang 43400, Selangor, Malaysia

**Keywords:** palm kernel cake, decanter cake, nutrient digestibility, poultry feed, standardized ileal digestibility, amino acids, fiber

## Abstract

This study aimed to evaluate the composition of palm kernel cake (PKC) and decanter cake (DC) and assess their nutrient digestibility in broilers. One hundred one-day-old male broiler chicks (Ross 308) were divided into two dietary treatment groups. The diets were 90.3% PKC- and DC-based and contained 0.3% titanium dioxide (TiO_2_) as an indigestible marker. The data were analyzed using the Statistical Analyses System (SAS). Treatment means were compared using a two-sample *t*-test at a 5% significance level. This study found that PKC had a higher (*p* < 0.05) crude protein (CP) content (18.19%) and lower (*p* < 0.05) ether extract (EE) content (9.33%) compared to DC (16.47% CP, 11.17% EE). Additionally, DC contained a higher (*p* < 0.05) ash, crude fiber (CF), and gross energy (GE) content. Both PKC and DC had the highest standard ileal digestibility (SID) for energy (EE), with PKC showing a higher SID (*p* < 0.05) for CP and EE compared to DC. The apparent metabolizable energy (AME) values were 2079.47 kcal/kg for PKC and 2011 kcal/kg for DC, with no significant differences (*p* > 0.05) between these by-products. Furthermore, glutamic acid was the most abundant amino acid (AA) in both PKC (45.12 g/kg) and DC (25.59 g/kg). Additionally, PKC had higher (*p* < 0.05) SID values for methionine (58.58%) and arginine (64.84%) compared to DC (33.15% and 14.09%), with arginine showing the highest SID in PKC (64.84%) and alanine in DC (46.48%).

## Simple Summary

Palm kernel cake (PKC) and decanter cake (DC) are by-products of the palm oil industry with the potential to be cost-effective feed ingredients for poultry. This study evaluated the nutritional composition of the feed and examined how well broiler chickens can digest the nutrients it provides. Young broiler chickens were fed diets primarily composed of either PKC or DC, and key nutrient digestibility was assessed. The analysis revealed that PKC contained more protein and less fat compared to DC and was more easily digested, particularly in terms of protein and fat. In contrast, DC had higher levels of fiber and overall energy. Glutamic acid was the most abundant amino acid in both by-products, while PKC showed superior digestibility for essential amino acids such as methionine and arginine. These findings highlight the nutritional value of both PKC and DC, with PKC demonstrating more favorable digestibility characteristics in terms of key essential amino acids for poultry. This knowledge can support the efficient use of agricultural by-products in poultry diets, contributing to more sustainable and economically viable poultry production systems.

## 1. Introduction

Feed costs constitute the largest expense in poultry production, prompting the exploration of locally available, low-cost alternatives, such as palm oil by-products, to reduce production costs. However, these by-products are often of lower quality due to their reduced digestibility and nutrient availability for poultry [1]. Palm oil industries produce significant by-products annually, including PKC and DC. PKC is derived from palm nuts using an expeller press or solvent oil extraction techniques. DC is the solid material remaining after decanter centrifugation, which separates oil from solids [2,3].

Nutrient digestibility in broiler chickens is crucial for feed efficiency, growth performance, and overall health [4]. Several factors affect how efficiently nutrients are digested, such as the type and amount of CF and anti-nutritional elements in the diet, which can reduce nutrient absorption. Additionally, other factors such as the age, physiological condition, and health status of animals can also affect nutrient absorption and utilization [5,6]. Furthermore, interactions between feed ingredients and the category of animal further influence variations in nutrient digestibility. For instance, the lack of specific enzymes required to break down fiber in the non-ruminant digestive system can adversely affect nutrient absorption and the utilization of ingredients with high fiber contents [1].

PKC at 15% inclusion levels in broiler diets decreased dry matter (DM), CP, EE, fibers, and ash apparent ileal digestibility (AID) [7]. Similarly, the digestibility of CP in broiler chickens was significantly lower for birds that received 10%, 20%, 30%, and 40% PKC compared to control-group birds [8]. Palm oil by-products, such as PKC, may contain significant amounts of anti-nutritional factors (ANFs), including non-starch polysaccharides (NSPs) [9]. The adverse effects of PKC on nutrient digestibility may be attributed to its high NSPs and fiber content, which increases the viscosity of digesta and accelerates its passage through the intestines [7,10,11]. Furthermore, mannan polysaccharide is a crucial constituent of NSPs in PKC, which is not digestible by the digestive enzymes of non-ruminant species. This causes a decrease in nutrient digestibility when PKC levels increase in poultry diets [1].

The digestibility of nutrients is measured in both standardized and apparent forms, which are essential measures of nutrient utilization efficiency. The SID of nutrients in birds refers to the proportion of a nutrient that is digested and absorbed by the bird in the ileum, considering basal endogenous losses (BELs) or secretions from the digestive tract [12,13]. The AID of nutrients does not consider BELs, but it may overestimate true digestion and absorption [14]. Regarding feed formulation accuracy, it has been demonstrated that the SID values are more additive than the AID values [6,12,15,16]. This experiment aimed to assess the nutrient composition and digestibility of PKC and DC, which are readily available by-products of the palm oil industry. These by-products offer potential as sustainable and cost-effective alternatives in broiler diets. However, there is limited information available, particularly on the SID of nutrients in PKC and DC. Therefore, this study is necessary to provide updated data and a better understanding of the nutritional value and SID of these ingredients for practical use in poultry nutrition.

## 2. Materials and Methods

### 2.1. Research Place and Ethical Form

The research was conducted in compliance with the ethical guidelines approved by the Universiti Putra Malaysia (UPM) Institutional Animal Care and Use Committee (R040/2024).

### 2.2. Palm Kernel Cake and Decanter Cake Preparation

The PKC and DC used in this study were sourced from Sime Darby Plantation, a sustainable palm oil production company (Sime Darby Plantation, Carey Island 42960, Jugra, Selangor, Malaysia).

### 2.3. Experimental Design and Diets

A total of one hundred one-day-old male broiler chickens (Ross 308) were obtained from a local hatchery (FFM Farms Sdn. Bhd., 47000 Sungai Buloh, Selangor, Malaysia). The birds were raised in a temperature- and humidity-controlled closed commercial poultry house, following the Ross 308 management guideline. The temperature was maintained at 32 ± 1 °C on day 1 and gradually lowered to approximately 24 ± 1 °C by the end of the feeding trial. The temperature was controlled based on the humidity levels in the poultry house. The average relative humidity during the experimental period was 70 ± 5%. Birds were provided with continuous lighting throughout the experimental period. The poultry house was equipped with a penning cage system (120 × 120 cm in length × width) with plastic mesh flooring elevated approximately 60 cm above the ground. Plastic sheets were placed on the ground beneath the cages, and cleaning was carried out on a weekly basis to maintain hygiene. The birds were vaccinated against Newcastle disease and Infectious Bronchitis at 8 and 21 days of age and against Infectious Bursal disease at 14 days of age. The birds were fed commercial feed from day 1 to 18, with ad libitum access to feed and water. The birds were randomly divided into two experimental dietary treatment groups, PKC- and DC-based (Table 1), with five replicates per treatment and ten birds per replicate (cage). The birds were given unrestricted access to the experimental diet and drinking water throughout the nine-day trial. Titanium dioxide (TiO_2_) was added to the experimental diets at a concentration of 0.3% to serve as an indigestible marker for assessing nutrient digestibility.

### 2.4. Sampling

At the end of the feeding trial, birds were transferred to the slaughterhouse of the animal science department, UPM, and euthanized in accordance with halal procedures, following the guidelines outlined in the Department of Standards Malaysia standard [17]. The digesta were collected from the Meckel’s diverticulum to a point about 1 cm proximal to the ileocecal junction. The digesta were gently squeezed into plastic pillboxes and freeze-dried.

The feed samples were collected after the feed ingredients were mixed. The samples were milled into a fine powder and kept at −20 °C until chemical analysis.

### 2.5. Chemical Analysis

#### 2.5.1. Proximate Analysis

The proximate analysis used the standard procedure of the Association of Official Analytical Chemists (AOAC) [18]. The weights of the CP, EE, and ash contents were subtracted from 100% to determine the total carbohydrate contents [100% − (% CP + % EE + % ash)] [19]. The acid detergent fiber (ADF), neutral detergent fiber (NDF), and acid detergent lignin (ADL) contents were determined using the method described by Van Soest [20]. The hemicellulose value was calculated by subtracting ADF from NDF, whereas cellulose was determined as ADF − ADL.

#### 2.5.2. Measurement of Gross Energy

The GE value was determined using the fully automated C300 basic combustion oxygen bomb calorimeter (IKA, Staufen, Germany). A 1 g pelletized sample was placed in the apparatus as described by Azizi et al. [14]. The heat output (GE) of a unit sample was automatically measured when it was completely burned to produce carbon dioxide and water, the products of its ultimate oxidation.

#### 2.5.3. Amino Acid Analysis

The amino acid profile was determined using High-Performance Liquid Chromatography (HPLC) with a fluorescence detector (Waters Alliance e2695 system, Waters Corporation, Milford, MA, USA). The procedure consisted of the main steps of hydrolysis, derivatization, chromatographic separation, and quantification.

Hydrolysis and derivatization: Approximately 0.2 g of the sample was hydrolyzed in 5 mL of 6N HCl at 110 °C for 24 h. A 4 mL volume of 2.5 mM L-2-aminobutyric acid was added as an internal standard. The hydrolysate was then diluted to 100 mL in a volumetric flask with ultrapure water. A 1.5 mL aliquot of the hydrolysate solution was filtered through a 0.45 µm PTFE syringe filter before analysis.

Derivatization was performed by mixing 70 µL of borate buffer with 10 µL of the filtered hydrolysate or standard solution, followed by the addition of 20 µL of freshly prepared AccQ-Fluor reagent (Waters Corporation, Milford, MA, USA). The mixture was vortexed and allowed to stand at room temperature for 1 min before being heated at 55 °C for 10 min. The derivatized samples were then transferred into vials for subsequent HPLC analysis.

Chromatographic conditions and quantification: Chromatographic separation was performed using a Waters AccQ-Tag column, 3.9 × 150 mm, 4 µm (Waters Corporation, Milford, MA, USA) maintained at a temperature of 36 ± 1 °C with a flow rate of 1 mL/min and an injection volume of 10 µL. The mobile phase system consisted of Eluent A (AccQ-Tag Eluent A diluted 1:10 with deionized water), Eluent B (100% acetonitrile), and Eluent C (100% ultrapure water). Amino acids were detected using a fluorescence detector set to an excitation wavelength of 250 nm and an emission wavelength of 395 nm.

Quantification was achieved through external standard calibration using a standard solution containing 2.5 mM of 16 amino acids (tyrosine, leucine, glutamic acid, isoleucine, methionine, valine, arginine, histidine, proline, phenylalanine, alanine, aspartic acid, glycine, lysine, threonine, serine), 1.25 mM cysteine, and 2.5 mM L-2-aminobutyric acid as the internal standard. Amino acid concentrations were calculated from the detected amounts (ng) using an internal standard for calibration. The percentage of each amino acid (% *w*/*w*) in the sample was calculated as follows: the weight percent (% *w*/*w*) of each amino acid in the sample = (amount of amino acid injected (ng)/total amount of amino acid injected (ng)) × 100%.

#### 2.5.4. Measurement of Titanium Dioxide

The method for determining titanium dioxide followed the procedure outlined by Short et al. [21], as described earlier [14]. Shortly, a 0.1 g portion of the feed and digesta were measured in pre-weighed crucibles and ashed at a temperature of 550 °C for seven hours. After cooling, 10 mL of 7.4 M sulfuric acid was added to each crucible, and the samples were gently heated on a hot plate for 60 min until completely dissolved. Subsequently, after cooling, the solutions were transferred to beakers with 25 mL distilled water and filtered through Whatman filter paper (No. 541) into 100 mL volumetric flasks. To each flask, 20 mL of 30% hydrogen peroxide was added, followed by topping up to 100 mL with distilled water. A 0.3 mg/mL TiO_2_ standard solution was prepared in concentrated sulfuric acid to generate varying-concentration standards. A blank sample without titanium dioxide was also prepared for reference. The absorbance of the sample solutions was measured using a Multiskan GO spectrophotometer (Thermo Scientific, Waltham, MA, USA) at 410 nm.

### 2.6. Calculations

#### 2.6.1. Calculations of AID, BELs, and SID

Calculations were performed using data expressed on a DM basis. The percentage of AID of nutrients was determined using the following formula:

AID (%) = [[(nutrient/TiO_2_)_diet_ − (nutrient/TiO_2_)_ileal digesta_]/(nutrient/TiO_2_)_diet_] × 100

The BEL flow was calculated as mg of nutrient flow per kg of the DM intake [12,22].

BEL flow (mg/kg) = nutrients in ileal digesta (mg/kg) × [TiO_2_ in diet (mg/kg)/TiO_2_ in digesta (mg/kg)]

The AID values for nutrients were standardized using the BELs to get the percentage of SID as follows:

SID (%) = AID (%) + [BEL flow (mg/kg)/Ingredients (mg/kg)]

#### 2.6.2. Calculation of Apparent Metabolizable Energy

The calculation of apparent metabolizable energy (AME) followed the procedure described by Scott and Boldaji [23], applying the equation below:

AME (kcal/kg) = GE value of diet − [GE value of digesta × (TiO_2_ in diet_/_TiO_2_ in digesta)]

### 2.7. Statistical Analysis

All data were analyzed using SAS software (version 9.4; SAS Institute Inc., Cary, NC, USA). As the study involved two treatment groups, differences between means were assessed using independent samples *t*-tests. Assumptions of normality and homogeneity of variances were tested using the Shapiro–Wilk and Levene’s tests, respectively. Significance between groups was considered at *p* < 0.05.

## 3. Results

### 3.1. Chemical Composition of Palm Kernel Cake and Decanter Cake

#### 3.1.1. Proximate Composition and Gross Energy Value

The nutrient composition, fiber fractions, and GE values of PKC and DC are presented in Table 2. The organic matter, CP, and carbohydrate contents were significantly higher (*p* < 0.05) in PKC compared to DC. Meanwhile, the ash and EE contents were significantly higher (*p* < 0.05) in DC than in PKC. At the same time, the GE value was also higher (*p* < 0.05) in DC than in PKC. Regarding the fiber content, the CF, ADF, and cellulose contents were significantly higher (*p* < 0.05) in DC than in PKC. In contrast, NDF, ADL, and hemicellulose contents were higher (*p* < 0.05) in PKC than in DC.

#### 3.1.2. Amino Acid Contents

The AA contents of PKC and DC are presented in Table 3. Among all amino acids, glutamic acid had the highest concentration in PKC (45.12 g/kg) and DC (25.59 g/kg). In PKC, arginine and aspartic acid were the amino acids with the next-highest concentrations, while in DC, aspartic acid and leucine were the next-most abundant. On the other hand, histidine (3.98 and 4.07 g/kg) and methionine (4.09 and 2.38 g/kg) were the amino acids present in the lowest concentrations in PKC and DC, respectively.

Meanwhile, threonine, valine, leucine, isoleucine, proline, alanine, tyrosine, and serine concentrations were significantly higher (*p* < 0.05) in DC compared to PKC. On the other hand, lysine, methionine, arginine, and glutamic acid concentrations were significantly higher (*p* < 0.05) in PKC compared to DC. No significant differences (*p* > 0.05) were observed between PKC and DC in phenylalanine, histidine, aspartic acid, and glycine concentrations.

### 3.2. Apparent and Standardized Ileal Nutrient Digestibility

#### 3.2.1. Proximate Nutrient Digestibility and Apparent Metabolizable Energy

The AID, SID, and AME of nutrients in broiler chickens fed PKC- and DC-based diets are presented in Table 4. Among the analyzed contents, EE recorded the highest AID and SID (above 90%) for PKC and DC. At the same time, the EE, CP, AID, and SID values were significantly higher (*p* < 0.05) for PKC compared to DC.

The AME values were 2079.47 kcal/kg and 2011.11 kcal/kg for PKC and DC, respectively, without significant differences (*p* > 0.05) between these raw materials.

#### 3.2.2. Amino Acid Digestibility

The AID and SID of AA in birds fed PKC- and DC-based diets are presented in Table 5. The AID and SID values varied among the AAs. Arginine recorded the highest AID (64.49%) and SID (64.84%) values among all AAs, followed by methionine (58.16 and 58.58%, respectively) and glutamic acid (58.12 and 58.53%, respectively) for the PKC-group birds. In addition, alanine showed the highest AID (45.93%) and SID (46.48%) values among the other AAs for the DC group, followed by aspartic acid (43.44% and 44.01%, respectively) and proline (43.13% and 43.70%, respectively). On the other hand, among the AAs for the PKC and DC groups, lysine displayed the lowest AID (6.66% and 7.60%, respectively) and SID (6.97% and 7.90%, respectively) values.

Additionally, methionine, arginine, and glutamic acid had significantly higher (*p* < 0.05) AID and SID values in the PKC group compared to the DC group. Conversely, the AID and SID values for aspartic acid and alanine were significantly higher (*p* < 0.05) in the DC group compared to the PKC group.

## 4. Discussion

### 4.1. Chemical Composition

#### 4.1.1. Proximate Nutrient Composition and Gross Energy

PKC and DC are documented as livestock feed components, fertilizers, and biogas production materials [24,25,26,27]. The results of the current study show that the CP contents of PKC and DC were 18.19% and 16.47%, respectively. These values for CP in PKC and DC are similar to those in earlier reports [28,29]. However, the CP contents of PKC and DC are considered low for young chicks [30,31]. The results of the present study showed that PKC and DC contained 9.33% and 11.17% EE, 5.20% and 13.56% ash, and 67.29% and 58.80% total carbohydrate contents, respectively. These findings are within the range previously reported for the EE contents of PKC and DC [32] and ash contents in PKC [33,34]. However, the ash content of DC in the current study was higher than that reported in a previous study, which stated that DC contains a 6.30% ash content.

In terms of the CF and fiber fractions, the results showed that PKC and DC contained 14.70% and 19.02% CF, 79.14% and 77.23% NDF, 41.26% and 53.19% ADF, and 13.50% and 10.78% ADL contents. The CF contents of PKC and DC determined in the present study are similar to those reported earlier [32,35]. However, there are differences between the current study’s results and previous reports in the NDA, ADF, ADL, cellulose, and hemicellulose contents of PKC and DC. For instance, the ADF content was reported to be lower in DC (17%), whereas the ADL values were reported to be higher in both PKC (17.3%) and DC (13.8%), compared to the results of the current study [1,32,35]. The variation in fiber content between the current study and previous reports for PKC and DC may be attributed to several factors, including differences in palm oil tree variety, oil extraction methods, and variations in analytical techniques used [24,25,26,27].

The CF, ADF, NDF, and ADL contents of palm oil by-products are higher compared to corn and soybean meal [10]. CF comprises various fiber fractions, including cellulose, hemicellulose, lignin, fructan, pectin, and cutin [36]. NDF represents the cell wall contents consisting of hemicellulose, cellulose, and lignin. NDF contents are insoluble in neutral detergent solutions and are not properly digestible in non-ruminants [37]. ADF consists of plant cell wall components and the residual material left after boiling a plant-source sample in an acid detergent solution. ADF consists mainly of cellulose, lignin, and other compounds such as heat-damaged protein and silica [36]. The fiber contents of palm oil by-products may be acceptable for ruminants. However, for non-ruminants, these values are considered high [7,30].

The quantification of the chemical energy present in feed is determined through its conversion into heat. This transformation involves the oxidation of the sample through combustion, with the resulting heat serving as a measure of the GE per unit feed weight. GE is the total energy present in the feed (in its highest form) that enters the body and is lost to the environment through digestion [14,37]. The GE values of PKC and DC recorded in the present study were 4577.22 and 4746.73 kcal/kg, respectively. At the same time, the GE values reported in previous studies were lower for PKC (4089.39 kcal/kg) [38,39] and DC (4158.70 kcal/kg) [40]. The current study revealed a higher GE value for DC than for PKC. The fact that DC has more crude fat than PKC may account for the greater GE value.

#### 4.1.2. Amino Acid Contents

In the current study, glutamic acid was the most abundant AA in PKC (45.12 g/kg) and DC (25.59 g/kg). In PKC, arginine and aspartic acid had the next-highest concentrations, while aspartic acid and leucine were the most abundant after glutamic acid in DC. The data showed that the lysine concentration was 6.91 and 5.84 g/kg in PKC and DC, respectively. Furthermore, the concentrations of methionine were 4.09 and 2.38 g/kg in PKC and DC, respectively, and the concentrations of arginine were 23.90 and 8.47 g/kg in PKC and DC, respectively. Meanwhile, among the essential AAs, the concentrations of threonine, valine, leucine, and isoleucine were higher in DC compared to PKC. In contrast, the concentration of lysine, methionine, and arginine was higher in PKC compared to DC.

The AA contents of PKC and DC reported in previous studies differ slightly from those in the current study. For instance, the lysin concentration was reported to be 3.7 g/kg in PKC [28] and 10.6 g/kg in DC [29]. Additionally, the methionine concentration was 2.2 in PKC [28] and 5.0 in DC [29]. Similarly, the arginine concentration was 16.0 in PKC [28] and 6.2 in DC [29]. However, no recently published reports are available to explain the AA contents of palm oil by-products, particularly DC.

### 4.2. Nutrient Digestibility

#### 4.2.1. Proximate Nutrient Digestibility and Apparent Metabolizable Energy

As explained earlier, the adjustment for BELs is the only significant distinction between apparent and standardized digestibility levels [6,12,15,16]. A lack of research on palm oil by-products’ AID and SID in broilers, especially DC, limits our ability to compare findings openly with previous studies.

In the present study, the AID and SID of EE were above 90% for both PKC and DC. EE may contain triglycerides, steroids, fatty acids, phospholipids, essential oils, glycolipids, waxes, fat-soluble vitamins, carotenes, and xanthophylls [36]. The level of fat inclusion in the diet and the composition of fat can influence fat digestibility [41]. The EE content of PKC and DC contains various fatty acids, including linolenic acid, linoleic acid, stearic acid, lauric acid, palmitic acid, myristic acid, oleic acid, and arachidic acid [32]. The AID values of CP for PKC and DC were 46.57% and 39.40%, respectively, in the current study. The SID values were 47.02% and 39.92%, respectively. These findings are lower than those in the previous report, which stated that the AID of CP was 57.92% for PKC in broiler chickens [28]. The difference in CP digestibility could be due to the chemical composition of the by-products and the experimental conditions [1]. CP contains AAs and amines, proteins and peptides, purines and pyridines, and other nitrogen substances [36]. The protein digestibility of PKC and DC is commonly poorer than that of soybean meal and corn [16].

The fiber content of palm oil by-products is considered high for livestock such as poultry and pigs, particularly when the by-products are included at a high level in their diets [7,30]. It is well known that the digestive system of poultry lacks enzymes for fiber digestion. Additionally, research has shown that a high fiber content in the diet might negatively affect the nutrient digestibility of monogastric animals [7]. High NSP and fiber contents in diets such as CF, ADF, and NDF increase the viscosity of digesta, accelerate their passage time through the intestines, and adversely affect digestion [7,10,11].

Metabolizable energy (ME) refers to the energy an animal can utilize for its metabolic functions. The ME provides a better understanding of the useful energy in the feed compared to GE; it does not consider the energy dissipated as heat [42]. The AME values were 2079.47 and 2011.11 kcal/kg for PKC and DC, respectively. In the current study, the AME value of PKC and DC for broiler chickens was higher than that of earlier studies. For instance, the AME value of PKC and DC for poultry was reported to be 1910.26 kcal/kg [43]. The alterations in the AME could be due to the fat content of the palm oil by-product, with a higher fat content resulting in higher AME [44].

#### 4.2.2. Amino Acid Digestibility

It is worth considering that rather than consisting of the total AA content of feed ingredients, poultry feed should be formulated based on the required AA digestibility and bioavailability [45]. Digestibility refers to the fraction of nutrients broken down and absorbed from the gastrointestinal lumen, measuring the amount of accessible nutrients that enter the bloodstream. Bioavailability is the proportion of digested and absorbed nutrients the body can use effectively for its metabolic and physiological functions [46,47].

Directly measuring AA bioavailability in poultry is a complex challenge, and as a result, it is typically estimated through the digestibility of AAs [48]. Ileal digesta contains substances from the gut, as well as cellular material that has been abraded from the gut wall [6,41]. To effectively address such corrections, SID incorporates a correction process that ensures that AID is standardized [49]. The AA digestibility of agricultural by-products, such as PKC, is usually lower than that of soybean meal and corn [50]. It is well known that the digestibility of AAs in all common commercial feed ingredients is less than 100%. It can significantly vary among different ingredients and samples of the same ingredient [48].

Our research findings indicated differences in digestibility values among the AAs. In the PKC group of birds, arginine had the highest SID (64.84%) among all AAs, followed by methionine (58.16%) and glutamic acid (58.12%). In contrast, for the DC-group birds, alanine demonstrated the highest SID (46.48%) among all AAs, followed by aspartic acid (44.01%) and proline (43.70%). Similar findings were reported in previous research, where the AID of arginine was the highest among all AAs in birds fed a PKC-based diet [50]. Arginine, one of the essential AAs in poultry nutrition, positively affects broiler production performance [51].

Our results demonstrated that lysine had the lowest SID values among all AAs for the PKC-group (7.60%) and DC-group birds (7.90%). Threonine in the PKC group (27.47%) and histidine in the DC group (32.93%) ranked as the second-lowest AAs in terms of SID values. Previous studies have also reported that the digestibility of different AAs varies within the same ingredient [15,50]. Lysine digestibility is particularly important in poultry nutrition and is typically lower than that of other AAs within the same ingredient [48]. No recently published studies report the SID of AAs in PKC and DC for broiler chickens, so we cannot directly compare our results with others. However, it is well known that due to factors such as protein quality, incomplete AA profiles, anti-nutritional factors, cell wall components, and high fiber content, the digestibility of these agricultural by-products is usually lower than that of common poultry feed ingredients like soybean meal and corn [46,48,52].

## 5. Conclusions

PKC and DC are by-products of palm oil extraction and processing. The findings of this study highlight the nutritional potential of PKC and DC as alternative feed ingredients for poultry. PKC demonstrates a higher CP content than DC, while DC contains greater amounts of ash, EE, CF, and GE. Additionally, glutamic acid was the most abundant AA in PKC and DC, while histidine and methionine were the lowest in both by-products. Despite the differences in nutrient composition, both by-products exhibit lower SID values relative to conventional feed ingredients such as soybean meal and corn. PKC shows superior SID for EE, CP, and essential amino acids, including methionine and arginine, among these agricultural by-products. In contrast, DC displays a higher SID for non-essential amino acids such as alanine and aspartic acid. Both PKC and DC demonstrate the highest SID for EE and the lowest SID for lysin contents among all nutrients. The AME values were 2079.47 kcal/kg for PKC and 2011 kcal/kg for DC, with no significant differences between the two by-products. These findings highlight the different composition and lower digestibility of PKC and DC compared to conventional feedstuffs such as corn and soybean meal. While their nutritional limitations exist, their potential as sustainable by-products could still contribute to the economic and environmental sustainability of poultry production. Future research could focus on optimizing the nutritional value of PKC and DC through processing techniques, fermentation, and enzymatic supplementation to enhance their digestibility and overall efficacy as alternative feed ingredients.

## Figures and Tables

**Table 1:** Experimental trial diet composition.

Components (%)	Treatment Groups	
	PKC ^1^	DC ^2^
PKC	90.30	0.00
DC	0.00	90.30
Palm oil	6.00	6.00
Calcium carbonate	1.70	1.70
Sodium chloride	0.40	0.40
Vitamin blend ^3^	0.50	0.50
Mineral blend ^4^	0.50	0.50
Choline chloride	0.30	0.30
Titanium dioxide	0.30	0.30
Total	100.00	100.00

^1^ PKC; palm kernel cake. ^2^ DC; decanter cake. ^3^ Content per kg: Vit E (90.00 g), Vit K3 (6.00 g), Vit B1 (7.00 g), Vit B2 (22.00 g), Vit B6 (12.00 g), Vit B12 (0.07 g), Vit A (35.00 MIU), Vit D3 (9.00 MIU), nicotinic acid (120.00 g), pantothenic acid (35.00 g), folic acid (3.00 g), and biotin (300.00 mg). ^4^ Content per kg: manganese (100.00 g), iron (80.00 g), zinc (80.00 g), copper (15.00 g), potassium (4.00 g), sodium (1.50 g), iodine (1.00 g), selenium (0.20 g), and cobalt (0.25 g).

**Table 2:** Nutrient contents, fiber fractions, and GE values of PKC and DC.

Nutrient	PKC ^1^	DC ^2^	*p*-Value
Dry matter %	96.22 ± 0.07	96.08 ± 0.01	0.3201
Organic matter %	91.03 ± 0.12 ^a^	82.51 ± 0.32 ^b^	0.0015
Ash %	5.20 ± 0.6 ^b^	13.56 ± 0.33 ^a^	0.0012
Ether extract %	9.33 ± 0.20 ^b^	11.17 ± 0.10 ^a^	0.0466
Crude protein %	18.19 ± 0.20 ^a^	16.47 ± 0.08 ^b^	0.0058
Crude fiber %	14.70 ± 0.34 ^b^	19.02 ± 0.15 ^a^	0.0106
Carbohydrate %	67.29 ± 0.35 ^a^	58.80 ± 0.33 ^b^	0.0015
Neutral detergent fiber %	79.14 ± 0.10 ^a^	77.23 ± 0.08 ^b^	<0.0001
Acid detergent fiber %	41.26 ± 0.24 ^b^	53.19 ± 0.27 ^a^	<0.0001
Acid detergent lignin %	13.50 ± 0.20 ^a^	10.78 ± 0.21 ^b^	0.0352
Hemicellulose %	37.88 ± 0.29 ^a^	24.04 ± 0.33 ^b^	<0.0001
Cellulose %	27.77 ± 0.13 ^b^	42.41 ± 0.32 ^a^	<0.0001
Gross energy (kcal/kg)	4577.22 ± 18.77 ^b^	4746.73 ± 7.81 ^a^	0.0290

^a,b^ Significant differences are indicated by means in the same row that have different superscripts (*p* < 0.05). The data is displayed as the mean ± standard error. ^1^ PKC: palm kernel cake. ^2^ DC: decanter cake.

**Table 3:** Amino acid contents of PKC and DC (g/kg of the sample).

Amino Acids	PKC ^1^	DC ^2^	*p*-Value
Essential amino acids			
Threonine	7.38 ± 0.04 ^b^	9.74 ± 0.04 ^a^	0.0029
Valine	12.41 ± 0.05 ^b^	13.42 ± 0.14 ^a^	0.0247
Leucine	15.55 ± 0.06 ^b^	17.91 ± 0.15 ^a^	0.0014
Phenylalanine	8.14 ± 0.04	8.64 ± 0.03	0.0562
Lysine	6.91 ± 0.07 ^a^	5.84 ± 0.05 ^b^	0.0017
Isoleucine	8.96 ± 0.03 ^b^	11.40 ± 0.02 ^a^	0.0012
Methionine	4.09 ± 0.01 ^a^	2.38 ± 0.04 ^b^	0.0012
Arginine	23.90 ± 0.12 ^a^	8.47 ± 0.03 ^b^	0.0002
Histidine	3.98 ± 0.01	4.07 ± 0.08	0.5019
Non-essential amino acids			
Proline	9.29 ± 0.13 ^b^	11.50 ± 0.03 ^a^	0.0067
Aspartic acid	20.06 ± 0.10	20.83 ± 0.14	0.2265
Alanine	11.26 ± 0.03 ^b^	14.70 ± 0.05 ^a^	0.0013
Tyrosine	4.46 ± 0.07 ^b^	5.84 ± 0.07 ^a^	0.0310
Serine	9.70 ± 0.06 ^b^	10.93 ± 0.03 ^a^	0.0174
Glycine	10.22 ± 0.07	10.95 ± 0.02	0.0515
Glutamic acid	45.12 ± 0.15 ^a^	25.59 ± 0.05 ^b^	0.0003

^a,b^ Significant differences are indicated by means in the same row that have different superscripts (*p* < 0.05). The data is displayed as the mean ± standard error. ^1^ PKC: palm kernel cake. ^2^ DC: decanter cake.

**Table 4:** Apparent and standardized digestibility of nutrients in broilers fed 90.3% PKC- and DC-based diets.

Content (%)	AID ^1^			SID ^2^		
	PKC	DC	*p*-Value	PKC	DC	*p*-Value
Dry matter	61.96 ± 0.59	63.28 ± 1.05	0.7517	62.29 ± 0.59	63.60 ± 1.04	0.7521
Organic matter	64.06 ± 0.65	67.86 ± 1.31	0.4019	64.36 ± 0.64	68.13 ± 1.30	0.4022
Ash	37.28 ± 1.42	39.79 ± 1.86	0.1521	37.82 ± 1.41	40.30 ± 1.84	0.1520
Ether extract	94.77 ± 0.12 ^a^	90.68 ± 0.30 ^b^	0.0074	94.82 ± 0.12 ^a^	90.76 ± 0.29 ^b^	0.0074
Crude protein	46.57 ± 0.51 ^a^	39.40 ± 0.44 ^b^	0.0157	47.02 ± 0.50 ^a^	39.92 ± 0.43 ^b^	0.0156
Crude fiber	36.97 ± 0.93	37.93 ± 1.23	0.3050	37.51 ± 0.92	38.46 ± 1.22	0.3047
Carbohydrate	17.10 ± 1.41	18.15 ± 1.27	0.3131	17.81 ± 1.39	18.84 ± 1.26	0.3129
Neutral detergent fiber	18.71 ± 1.26	17.79 ± 1.80	0.7839	19.40 ± 1.25	18.49 ± 1.78	0.7839
Acid detergent fiber	20.18 ± 0.81	19.48 ± 1.23	0.6856	20.86 ± 0.80	20.17 ± 1.27	0.6857
Acid detergent lignin	13.43 ± 1.04	15.18 ± 0.78	0.2215	14.17 ± 1.03	15.90 ± 0.77	0.2214
Metabolizable energy (kcal/kg)	2079.47 ± 44.33	2011.11 ± 37.9	0.3019	-	-	-

^a,b^ Significant difference is shown by means in the same row with distinct superscripts (*p* < 0.05). The data is displayed as the mean ± standard error. ^1^ AID: apparent ileal digestibility. ^2^ SID: standardized ileal digestibility. PKC: palm kernel cake. DC: decanter cake.

**Table 5:** Apparent and standardized digestibility of amino acids in broilers fed 90.3% PKC- and DC-based diets.

Content	AID ^1^			SID ^2^		
	PKC	DC	*p*-Value	PKC	DC	*p*-Value
Essential amino acids						
Threonine	26.74 ± 1.60	36.32 ± 1.44	0.1497	27.47 ± 1.59	36.96 ± 1.43	0.1504
Valine	48.62 ± 0.67	41.30 ± 1.31	0.0533	49.13 ± 0.67	41.89 ± 1.29	0.0530
Leucine	44.42 ± 0.83	40.26 ± 1.27	0.2069	44.97 ± 0.82	40.86 ± 1.26	0.2068
Phenylalanine	50.34 ± 1.53	39.86 ± 1.14	0.0936	50.83 ± 1.51	40.46 ± 1.13	0.0936
Lysine	6.66 ± 1.51	6.97 ± 0.81	0.6069	7.60 ± 1.50	7.90 ± 0.81	0.6067
Isoleucine	42.33 ± 0.81	41.08 ± 1.24	0.5168	42.91 ± 0.80	41.67 ± 1.23	0.5164
Methionine	58.16 ± 0.27 ^a^	32.48 ± 1.81 ^b^	0.0179	58.58 ± 0.27 ^a^	33.15 ± 1.79 ^b^	0.0179
Arginine	64.49 ± 0.93 ^a^	13.23 ± 1.43 ^b^	0.0040	64.84 ± 0.92 ^a^	14.09 ± 1.42 ^b^	0.0040
Histidine	37.33 ± 0.86	32.26 ± 2.02	0.3808	37.95 ± 0.86	32.93 ± 2.01	0.3813
Non-essential amino acids						
Proline	37.79 ± 1.64	43.13 ± 1.43	0.0082	38.32 ± 1.63	43.70 ± 1.41	0.0082
Aspartic acid	31.99 ± 0.88 ^b^	43.44 ± 0.74 ^a^	0.0028	32.67 ± 0.87 ^b^	44.01 ± 0.74 ^a^	0.0028
Alanine	39.59 ± 0.89 ^b^	45.93 ± 0.63 ^a^	0.0046	40.19 ± 0.62 ^b^	46.48 ± 0.88 ^a^	0.0046
Tyrosine	40.05 ± 0.31	37.53 ± 0.76	0.0801	40.66 ± 0.31	38.16 ± 0.75	0.0790
Serine	39.54 ± 1.54	38.78 ± 1.36	0.7211	40.15 ± 1.52	39.39 ± 1.34	0.7200
Glycine	39.30 ± 1.79	40.25 ±1.25	0.6726	39.90 ± 1.78	40.84 ± 1.24	0.6734
Glutamic acid	58.12 ± 1.52 ^a^	41.26 ± 1.15 ^b^	0.0049	58.53 ± 0.51 ^a^	41.85 ± 1.14 ^b^	0.0049

^a,b^ Significant difference is shown by means in the same row with distinct superscripts (*p* < 0.05). The data is displayed as the mean ± standard error. ^1^ AID: apparent ileal digestibility. ^2^ SID: standardized ileal digestibility. PKC: palm kernel cake. DC: decanter cake.

## Data Availability

The data and materials that support the findings of this study are comprehensively presented in this article.

## Abbreviations

The abbreviations listed below are employed in this manuscript:

AA	Amino Acid
ADF	Acid Detergent Fiber
ADL	Acid Detergent Lignin
AID	Apparent Ileal Digestibility
AME	Apparent Metabolizable Energy
ANFs	Anti-Nutritional Factors
AOAC	Association of Official Analytical Chemists
BELs	Basal Endogenous Losses
CF	Crude Fiber
CP	Crude Protein
DC	Decanter Cake
DM	Dry Matter
EE	Ether Extract
GE	Gross Energy
GLM	General Linear Model
HPLC	High-Performance Liquid Chromatography
IACUC	Institutional Animal Care and Use Committee
ME	Metabolizable Energy
NDF	Neutral Detergent Fiber
NSP	Non-Starch Polysaccharides
PKC	Palm Kernel Cake
SAS	Statistical Analysis System
SID	Standardized Ileal Digestibility
TiO_2_	Titanium Dioxide

## References

[1] Azizi M.N., Loh T.C., Foo H.L., Chung E.L.T. (2021). Is palm kernel cake a suitable alternative feed ingredient for poultry?. Animals.

[2] Abioye K.J., Harun N.Y., Sufian S., Yusuf M., Kamyab H., Hassan M.A., Jagaba A.H., Sikiru S., Ubaidullah M., Pandit B. (2023). Regulation of ash slagging behavior of palm oil decanter cake by alum sludge addition. Chemosphere.

[3] Maniam G.P., Hindryawati N., Nurfitri I., Jose R., Mohd M.H., Dahalan F.A., Yusoff M.M. (2013). Decanter cake as a feedstock for biodiesel production: A first report. Energy Convers. Manag..

[4] Azizi M.N., Loh T.C., Foo H.L., Izuddin W.I. (2024). Growth performance, apparent ileal digestibility, and nutrient transporter gene expressions of broilers fed seaweed-supplemented diets. Trop. Anim. Sci. J..

[5] Barua M., Abdollahi M.R., Zaefarian F., Wester T.J., Girish C.K., Chrystal P.V., Ravindran V. (2021). Basal ileal endogenous amino acid flow in broiler chickens as influenced by age. Poult. Sci..

[6] Stein H.H., Sève B., Fuller M.F., Moughan P.J., De Lange C.F.M. (2007). Invited review: Amino acid bioavailability and digestibility in pig feed ingredients: Terminology and application. J. Anim. Sci..

[7] Alshelmani M.I., Loh T.C., Foo H.L., Sazili A.Q., Lau W.H. (2016). Effect of feeding different levels of palm kernel cake fermented by Paenibacillus polymyxa ATCC 842 on nutrient digestibility, intestinal morphology, and gut microflora in broiler chickens. Anim. Feed Sci. Technol..

[8] Aya V.E., Ayanwale B.A., Ijaiya A.T., Aremu A. (2013). Performance and nutrient digestibility in broiler chicks as influenced by multienzyme addition to starter diets containing palm kernel meal. Biotechnol. Anim. Husb..

[9] Usman Zamani H., Chwen Loh T., Ling Foo H., Samsudin A., Alshelmani M. (2017). Effects of Feeding Palm Kernel Cake with Crude Enzyme Supplementation on Growth Performance and Meat Quality of Broiler Chicken. Int. J. Microbiol. Biotechnol..

[10] Sulabo R.C., Ju W.S., Stein H.H. (2013). Amino acid digestibility and concentration of digestible and metabolizable energy in copra meal, palm kernel expellers, and palm kernel meal fed to growing pigs. J. Anim. Sci..

[11] Son A.R., Hyun Y., Htoo J.K., Kim B.G. (2014). Amino acid digestibility in copra expellers and palm kernel expellers by growing pigs. Anim. Feed Sci. Technol..

[12] Barua M., Abdollahi M.R., Zaefarian F., Wester T.J., Girish C.K., Ravindran V. (2020). Standardized ileal amino acid digestibility of protein sources for broiler chickens is influenced by the feed form. Poult. Sci..

[13] Cowieson A.J., Sorbara J.O.B., Pappenberger G., Abdollahi M.R., Ravindran V. (2020). Toward standardized amino acid matrices for exogenous phytase and protease in corn–soybean meal–based diets for broilers. Poult. Sci..

[14] Azizi M.N., Loh T.C., Foo H.L., Akit H., Izuddin W.I., Shazali N., Teik Chung E.L., Samsudin A.A. (2021). Chemical compositions of brown and green seaweed, and effects on nutrient digestibility in broiler chickens. Animals.

[15] Cowieson A., Sorbara J.O., Pappenberger G., Abdollahi M.R., Roos F.F., Ravindran V. (2019). Additivity of apparent and standardized ileal amino acid digestibility of corn and soybean meal in broiler diets. Poult. Sci..

[16] Kong C., Adeola O. (2013). Additivity of amino acid digestibility in corn and soybean meal for broiler chickens and White Pekin ducks. Poult. Sci..

[17] (2019). Halal Food—General Requirements (Third Revision).

[18] Latimer G.W., AOAC International (2005). Official Methods of Analysis of AOAC International.

[19] Edwards R.A., McDonald P., Greenhalgh J.F.D., Morgan C.A., Sinclair L.A., Wilkinson R.G. (2010). Animal Nutrition. Nature.

[20] Soest P.V. (1963). Use of Detergents in the Analysis of Fibrous Feeds. II. A Rapid Method for the Determination of Fiber and Lignin. J. Assoc. Off. Agric. Chem..

[21] Short F.J., Gorton P., Wiseman J., Boorman K.N. (1996). Determination of titanium dioxide added as an inert marker in chicken digestibility studies. Anim. Feed Sci. Technol..

[22] Moughan P.J., Marlies Leenaars G.S. (1992). Endogenous amino acid flow in the stomach and small intestine of the young growing pig. J. Sci. Food Agric..

[23] Scott T.A., Boldaji F. (1997). Comparison of Inert Markers [Chromic Oxide or Insoluble Ash (Celite^TM^)] for Determining Apparent Metabolizable Energy of Wheat- or Barley-Based Broiler Diets with or without Enzymes. Poult. Sci..

[24] Chusut W., Kanchanasuta S., Inthorn D. (2023). Optimization for biohydrogen purification process by chemical absorption techniques. Sustain. Environ. Res..

[25] Kanchanasuta S., Pisutpaisal N. (2016). Waste utilization of palm oil decanter cake on biogas fermentation. Int. J. Hydrogen Energy.

[26] Husin A.H., Hamzani S.H., Amirnordin S.H., Batcha M.F.M., Wahidon R., Wae-hayee M. (2022). Drying Studies of Oil Palm Decanter Cake for Production of Green Fertilizer. J. Adv. Res. Fluid Mech. Therm. Sci..

[27] e Silva A.R.B., Franzini V.I. (2024). Potential of organic wastes typical of the Brazilian Amazon for fertilizer use in agriculture. Environ. Chall..

[28] Alshelmani M.I., Loh T.C., Foo H.L., Sazili A.Q., Lau W.H. (2017). Effect of feeding different levels of palm kernel cake fermented by Paenibacillus polymyxa ATCC 842 on broiler growth performance, blood biochemistry, carcass characteristics, and meat quality. Anim. Prod. Sci..

[29] Djulardi A., Nuraini N., Trisna A. (2018). Palm oil sludge fermented with lentinus edodes in the diet of broilers. Int. J. Poult. Sci..

[30] Stein H.H., Casas G.A., Abelilla J.J., Liu Y., Sulabo R.C. (2015). Nutritional value of high fiber co-products from the copra, palm kernel, and rice industries in diets fed to pigs. J. Anim. Sci. Biotechnol..

[31] Lawal T.E., Lyayi E.A., Adeniyi B.A., Adaramoye O.A. (2010). Biodegradation of palm kernel cake with multienzyme complexes from fungi and its feeding value for broilers. Int. J. Poult. Sci..

[32] Abubakr A., Alimon A.R., Yaakub H., Abdullah N., Ivan M. (2015). Effect of feeding palm oil by-products based diets on muscle fatty acid composition in goats. PLoS ONE.

[33] Fadil M., Alimon A.R., Meng G.Y., Ebrahimi M., Farjam A.S. (2014). Palm kernel cake as a potential ingredient in Muscovy ducks diet. Ital. J. Anim. Sci..

[34] Akinyeye R.O., Adeyeye E.I., Fasakin O., Agboola A. (2011). Physico-chemical properties and anti-nutritional factors of palm fruit products (elaeis guineensis jacq.) from ekiti state nigeria. Electron. J. Environ. Agric. Food Chem..

[35] Abdul Razak M.N., Ibrahim M.F., Yee P.L., Hassan M.A., Abd-Aziz S. (2012). Utilization of oil palm decanter cake for cellulase and polyoses production. Biotechnol. Bioprocess Eng..

[36] Wu G. (2017). Principles of Animal Nutrition.

[37] Saha S.K., Pathak N.N. (2021). Fundamentals of Animal Nutrition.

[38] Alimon A.R. (2004). The Nutritive Value of Palm Kernel Cake for Animal Feed. Palm Oil Dev..

[39] Hakim A.H., Zulkifli I., Soleimani Farjam A., Awad E.A., Abdullah N., Chen W.L., Mohamad R. (2020). Passage time, apparent metabolisable energy and ileal amino acids digestibility of treated palm kernel cake in broilers under the hot and humid tropical climate. Ital. J. Anim. Sci..

[40] Abioye K.J., Harun N.Y., Umar H.A., Kolawole A.H. (2023). Study of Physicochemical Properties of Palm Oil Decanter Cake for Potential Syngas Generation. Chem. Eng. Trans..

[41] Blair R. (2008). Nutrition and Feeding of Organic Poultry.

[42] Scanes C.G., Christensen K.D. (2020). Poultry Science.

[43] Alimon A.R. (2009). Alternative Raw Materials for Animal Feed. War. Indones. Bull. Anim. Vet. Sci..

[44] Tang X. (2021). The nutritive value of palm kernel cake and its application in low quality diets of broiler chickens. Pakistan J. Agric. Sci..

[45] Ravindran V. (2021). Progress in ileal endogenous amino acid flow research in poultry. J. Anim. Sci. Biotechnol..

[46] Barua M., Abdollahi M.R., Zaefarian F., Wester T.J., Girish C.K., Chrystal P.V., Ravindran V. (2024). Effect of age on the standardized ileal amino acid digestibility of soybean meal and canola meal in broilers. Anim. Nutr..

[47] Selle P.H., Macelline S.P., Chrystal P.V., Liu S.Y. (2023). A reappraisal of amino acids in broiler chicken nutrition. Worlds. Poult. Sci. J..

[48] Parsons C.M. (2020). Unresolved issues for amino acid digestibility in poultry nutrition. J. Appl. Poult. Res..

[49] Lemme A., Ravindran V., Bryden W.L. (2004). Ileal digestibility of amino acids in feed ingredients for broilers. Worlds. Poult. Sci. J..

[50] Alshelmani M.I., Loh T.C., Foo H.L., Sazili A.Q., Lau W.H. (2017). Effect of solid state fermentation on nutrient content and ileal amino acids digestibility of palm kernel cake in broiler chickens. Indian J. Anim. Sci..

[51] Danish F., Amarkhil R., Jan A.N., Azizi M.N., Nasratullah H. (2023). Dietary arginine as a growth promoter for broiler chickens. Nangarhar Univ. Int. J. Biosci..

[52] Liu Y., Liu Y., Cao Y., Wang C. (2025). Pretreatment of Palm Kernel Cake by Enzyme-Bacteria and Its Effects on Growth Performance in Broilers. Animals.

